# Surface Modification of Erythrocytes with Lipid Anchors: Structure–Activity Relationship for Optimal Membrane Incorporation, in vivo Retention, and Immunocompatibility

**DOI:** 10.1002/anbr.202200037

**Published:** 2022-07-19

**Authors:** Hanmant Gaikwad, Guankui Wang, Yue Li, David Bourne, Dmitri Simberg

**Affiliations:** Translational Bio-Nanosciences Laboratory, Department of Pharmaceutical Sciences, The Skaggs School of Pharmacy and Pharmaceutical Sciences, University of Colorado Anschutz, Medical Campus, Aurora, CO 80045, USA; Colorado Center for Nanomedicine and Nanosafety, University of Colorado Anschutz Medical Campus, Aurora, CO 80045, USA; Translational Bio-Nanosciences Laboratory, Department of Pharmaceutical Sciences, The Skaggs School of Pharmacy and Pharmaceutical Sciences, University of Colorado Anschutz, Medical Campus, Aurora, CO 80045, USA; Colorado Center for Nanomedicine and Nanosafety, University of Colorado Anschutz Medical Campus, Aurora, CO 80045, USA; Translational Bio-Nanosciences Laboratory, Department of Pharmaceutical Sciences, The Skaggs School of Pharmacy and Pharmaceutical Sciences, University of Colorado Anschutz, Medical Campus, Aurora, CO 80045, USA; Colorado Center for Nanomedicine and Nanosafety University of Colorado Anschutz Medical Campus Aurora, CO 80045, USA; Center for Translational Pharmacokinetics and Pharmacogenomics, The Skaggs School of Pharmacy and Pharmaceutical Sciences, University of Colorado Anschutz Medical Campus, Aurora, CO 80045, USA; Translational Bio-Nanosciences Laboratory, Department of Pharmaceutical Sciences, The Skaggs School of Pharmacy and Pharmaceutical Sciences, University of Colorado Anschutz, Medical Campus, Aurora, CO 80045, USA; Colorado Center for Nanomedicine and Nanosafety, University of Colorado Anschutz Medical Campus, Aurora, CO 80045, USA

**Keywords:** complements, erythrocytes, half life, lipids, PEG, red blood cells, serum

## Abstract

Red blood cells (RBCs) are natural carriers for sustained drug delivery, imaging, and in vivo sensing. One of the popular approaches to functionalize RBCs is through lipophilic anchors, but the structural requirements for anchor stability and in vivo longevity remain to be investigated. Using fluorescent lipids with the same cyanine 3 (Cy3) headgroup but different lipid chain and linker, the labeling efficiency of RBCs and in vivo stability are investigated. Short-chain derivatives exhibited better insertion efficiency, and mouse RBCs are better labeled than human RBCs. Short-chain derivatives demonstrate low retention in vivo. Derivatives with ester bonds are especially unstable, due to removal and degradation. On the other hand, long-chain, covalently linked derivatives show remarkably long retention and stability (over 80 days half life in the membrane). The clearance organs are liver and spleen with evidence of lipid transfer to the liver sinusoidal endothelium. Notably, RBCs modified with PEGylated lipid show decreased macrophage uptake. Some of the derivatives promote binding of antibodies in human plasma and mouse sera and modest increase in complement deposition and hemolysis, but these do not correlate with in vivo stability of RBCs. Ultra-stable anchors can enable functionalization of RBCs for drug delivery, imaging, and sensing.

## Graphical Abstract

There is a great interest in surface modifications of red blood cells with lipid anchors for biomedical applications, but the stability of modifications is a problem. A library of fluorescent lipids is screened for stable incorporation into human and mouse RBCs. The modified RBCs are then injected in mice and tested for stability of the lipid and the circulation properties. The structural lipid requirements to maintain RBC stability and lower the immune recognition is thus investigated.

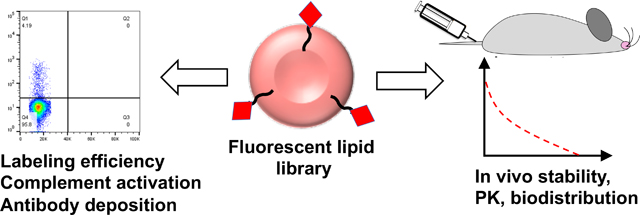

## 1. Introduction

Red blood cells (RBCs) are Mother Nature-made carriers of oxygen.^[1]^ Due to biocompatibility and in vivo longevity (up to 40 days life span in mice and 115 days in humans), RBCs have been explored for delivery of genes, chemotherapy, contrast agents, and enzymes.^[2]^ Several strategies to append molecules, enzymes, and nanoparticles to the RBC surface have been tested, including covalent modifications,^[3]^ targeting of integral membrane proteins,^[4]^ physical absorption,^[5]^ and genetic modification of progenitor cells.^[6]^ Each one of these approaches has advantages and disadvantages.^[7]^ Many groups, including ours, have been working on surface modification of RBCs with lipid anchors,^[8]^ due to the simplicity and versatility of the approach. We found that indocarbocyanine lipid DiI (dioctadecyl-Cy3 or DiI-C18), which is commonly used for labeling of cell membranes,^[9]^ showed much better retention in the RBC membrane than phospholipids, with up to 90% of the lipid present in the RBC membrane 48 h postinjection in mice.^[8a]^ We further prepared an aminomethyl derivative of DiI and conjugated it to thiolated enzymes and antibodies. The DiI-linked molecules efficiently painted mouse RBCs and showed good in vivo stability and retention.^[10]^

At the same time, our previous studies^[8a,10,11]^ explored a limited chemical space, and the retention of the anchor and the circulation of modified RBCs were followed only for a relatively short time (up to 5 days). An open question remains as to which structural parameters of the anchor determine the incorporation efficiency and retention. As mouse erythrocyte has a life span of ≈40 days,^[12]^ it would be interesting to observe the retention of the anchor over the entire life span. To address these questions, we prepared a library of lipid derivatives with the same Cy3 headgroup, but with different linkages between the headgroup and the lipophilic part, and different lipid chain types. The presence of the same fluorophore makes the comparison by flow cytometry straightforward and convenient. The results define the role of lipid structure in membrane incorporation and retention and open an avenue for stable functionalization of RBCs and adoptive cell therapies.

## 2. Results

### 2.1. Lipid Library Design and Incorporation Efficiency in RBC Membrane

**Figure 1.**
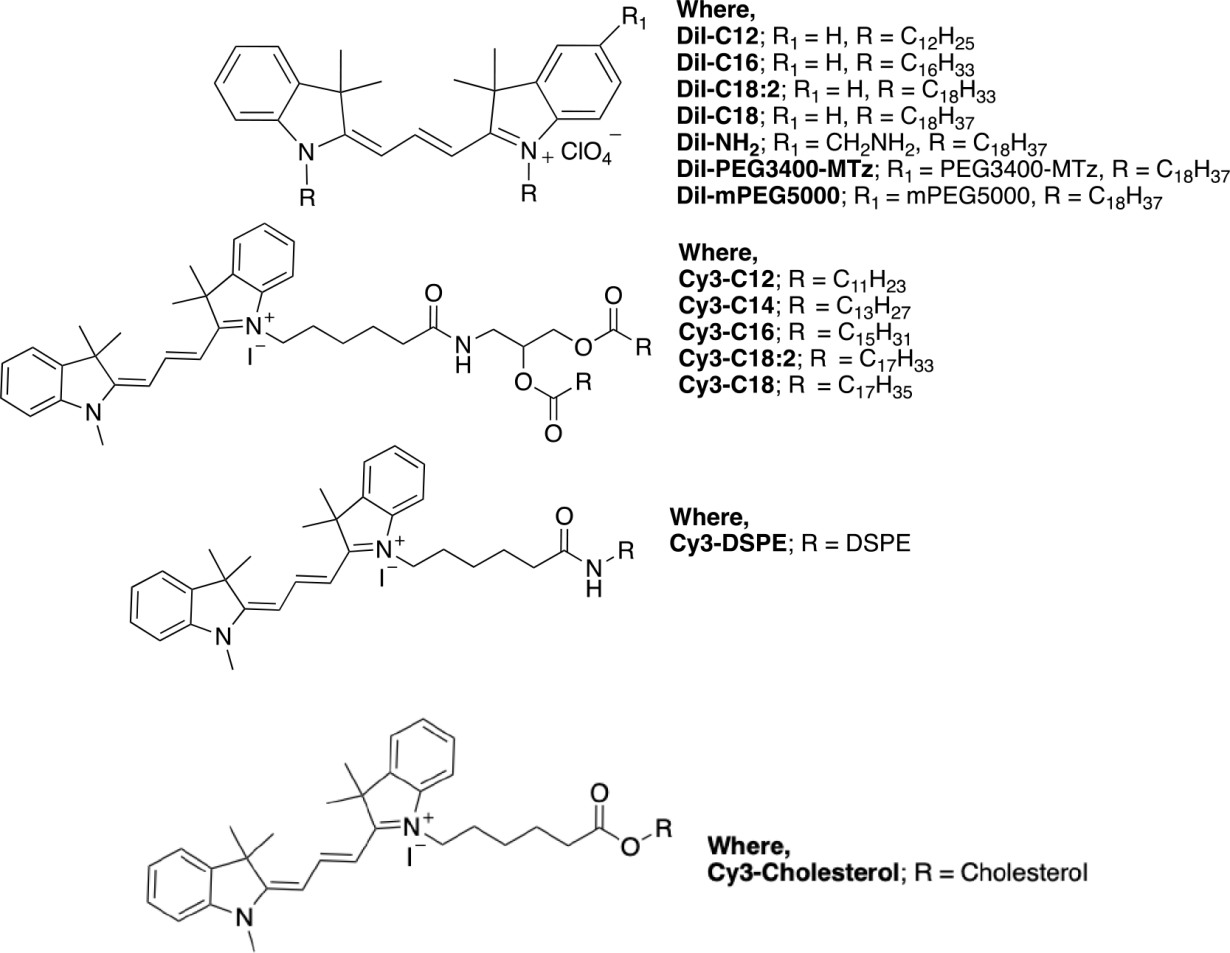
Structures of Cy3 derivatives used in the study.

**Figure 2.**
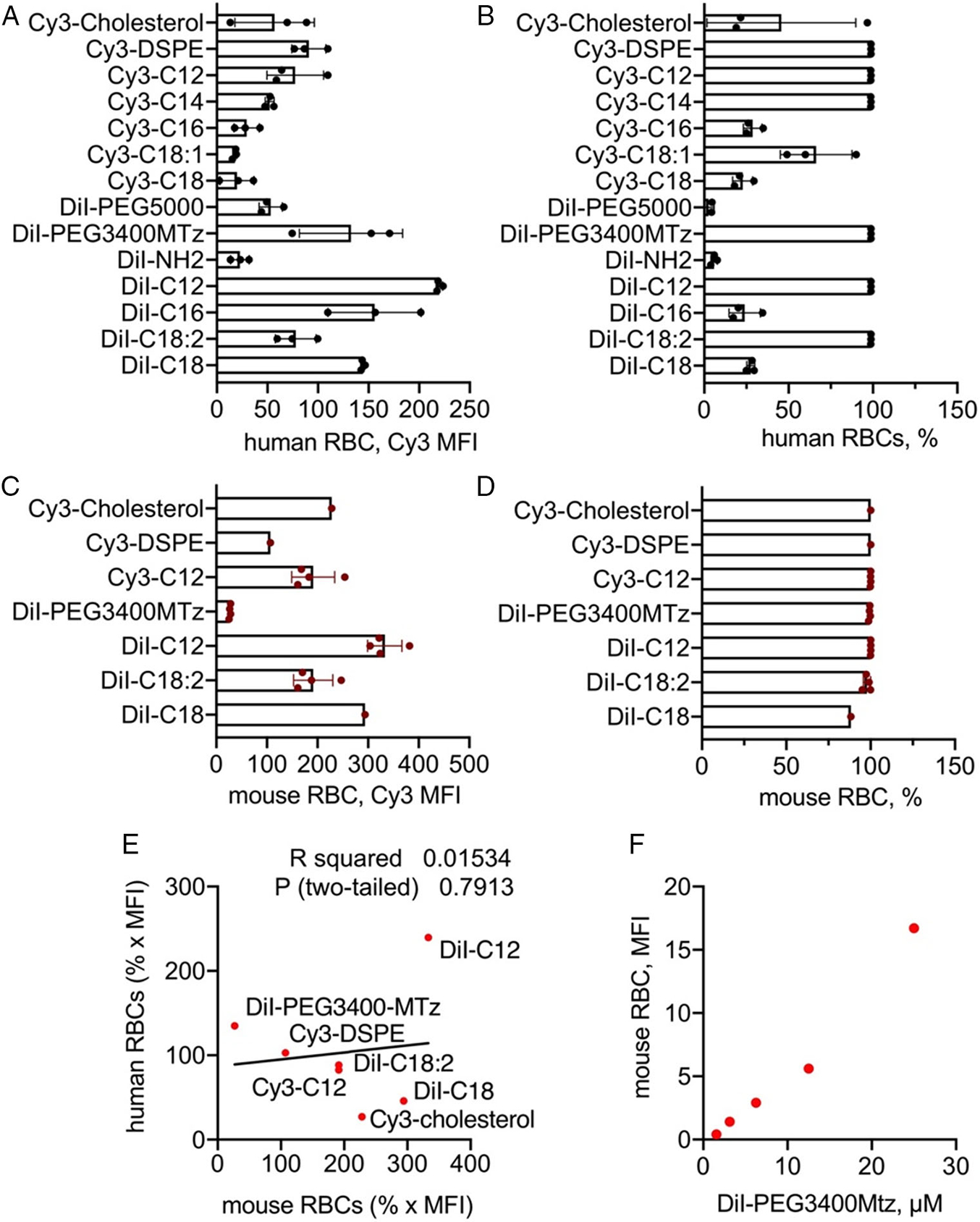
A–B) Labeling efficiency (MFI) and percentage of labeled human RBCs with derivates described in Figure 1. Data are means and SD of three healthy blood donors (male 45yo, male 67yo, and female 47yo). C–D) Labeling efficiency (MFI) and percentage of labeled mouse RBCs with derivatives used for in vivo study. Data are means and SD of 1–3 BALB/c-derived RBCs batches. E) Lack of correlation between mouse and human RBC labeling efficiency (MFI multiplied by the fraction of labeled RBCs (1 = 100%)). No correlation was observed for most lipids, except for DiI-C12 that showed the highest labeling of both mouse and human RBCs. F) Linearity of MFI of mouse RBCs labeled with different concentrations of DiI-PEG3400Mtz.

The lipid derivatives used in the study are shown in Figure 1. We acquired commercially available dialkyl indocarbocyanine lipids DiI-C18, DiI-C18:2, DiI-C16, DiI-C12 and synthesized additional headgroup derivatives of DiI-C18: DiI-amine, DiI-PEG5000, and DiI-PEG3400-methyltetrazine (Mtz). In addition, we prepared diacyl glycerol derivatives of Cy3: Cy3-C18, Cy3-C18:1, Cy3-C16, Cy3-C14, Cy3-C12, phospholipid derivative Cy3-distearoyl phosphatidylethanolamine (Cy3-DSPE), and Cy3-cholesterol. This library covers different lipid lengths, lipid types, and also headgroup-tail linkages. In addition, the library includes a limited number of headgroup derivatives relevant to drug delivery. Thus, PEGylated lipids have importance for erythrocyte PEGylation for blood camouflaging. Mtz is a click chemistry group that enables versatile modifications with biomolecules,^[13]^ but also serves as a mimic of a small molecule payload (*M*_w_ 230 Da). To determine the labeling efficiency, we used fresh human RBCs from two healthy donors. RBCs were incubated with lipids at 25 μM for 1 h and washed and analyzed with flow cytometry for the percentage of labeled cells and mean fluorescence intensity (MFI). Short-chain DiI-C12 and Cy3 diacyl glycerol (C12, C14) and long chain DiI-PEG5000, DiI-PEG3400Mtz, DiI-C18:2, and Cy3-DSPE resulted in the highest percentage of labeled human RBCs (Figure 2A). DiI-C12, DiI-C16, DiI-C18, Cy3-DSPE, and DiI-C18-PEG3400Mtz showed 2–3-fold higher MFI than the rest of the lipids (Figure 2B). Interestingly, amino DiI-C18 showed ≈4-fold lower labeling efficiency than the parent DiI-C18. Select lipids were then used to label mouse RBCs. The data in Figure 2C,D show generally higher mouse RBC labeling efficiency (MFI and percent RBCs) than human RBCs. There was no correlation between mouse and human RBC labeling efficiency, except DiI-C12, which exhibited the highest efficiency in both types of erythrocytes (Figure 2E). Finally, we checked the linearity of the Cy3 group signal when incorporated in RBCs. DiI-PEG3400Mtz showed linear increase in mouse RBC MFI with increasing labeling concentrations (Figure 2F).

**Figure 3.**
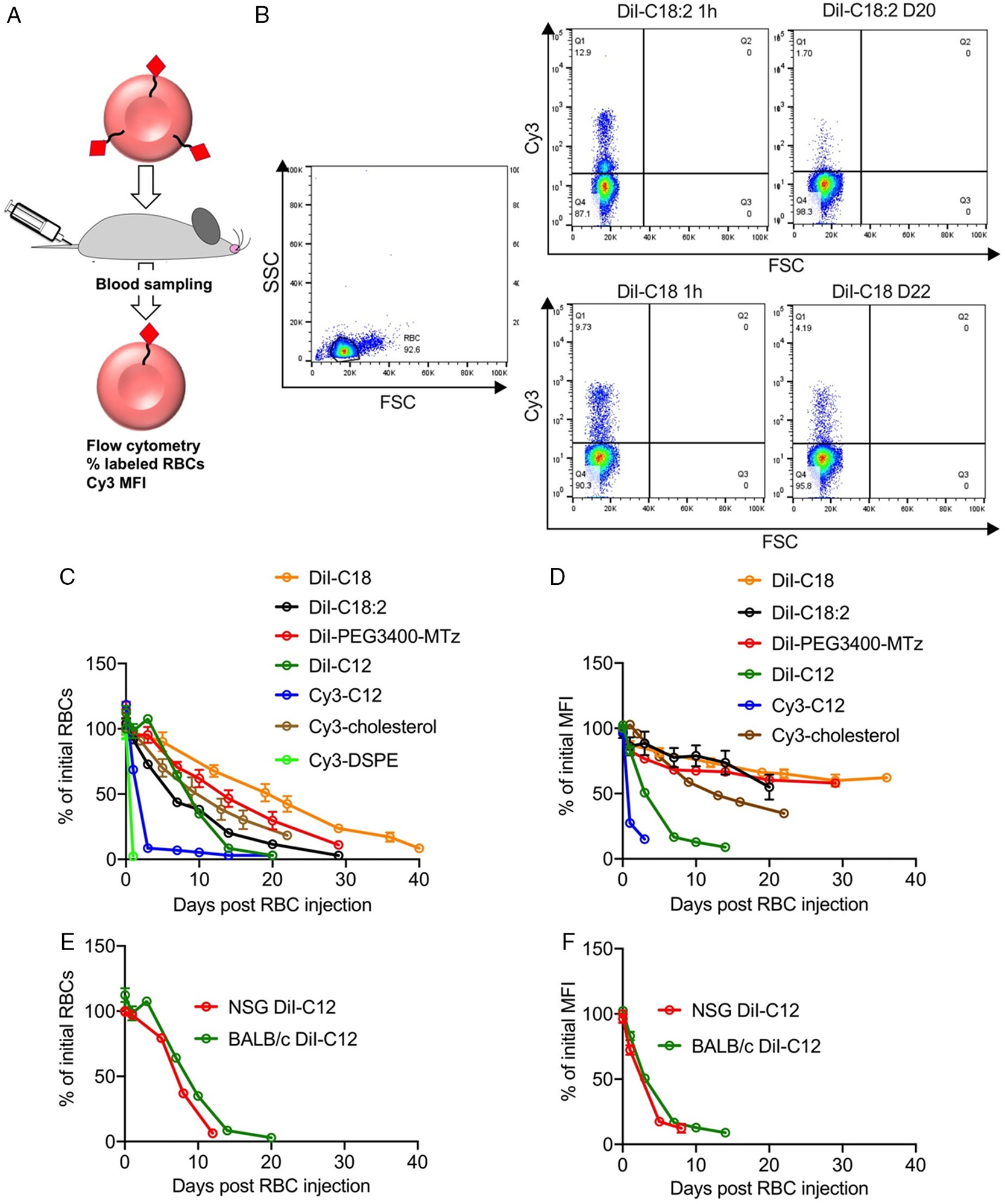
In vivo circulation properties and lipid retention in RBCs. A) Scheme of the experiment. B) Gating strategy and flow cytometry analysis of labeled RBCs (two time points are shown, for DiI-C18 and DiI-C18:2). C) Percent of injected RBCs (1 min postinjection = 100%). D) Percent of initial MFI of labeled RBCs (upper left quadrant in B; 1 min postinjection = 100%). E,F) %RBCs and %MFI for DiI-C12 injected in immunocompetent BALB/c and immunodeficient NSG mice. *N* = 3/group for all experiments.

Next, we investigated in vivo retention and longevity of modified RBCs. We used the lipids with the highest labeling efficiency (Figure 2C,D) for in vivo studies: DiI-C18, DiI-C18:2, DiI-C12, Cy3-C12, DiI-PEG3400Mtz, Cy3-DSPE, and Cy3-cholesterol. Labeled RBCs were injected intravenously in BALB/c mice and the circulating levels (percent positive RBCs) and the stability of the lipid (MFI of the labeled population) were analyzed by flow cytometry (Figure 3A). Representative dot plots for DiI-C18 and DiI-C18:2 (Figure 3B) show a distinct population of labeled RBCs at both 1 min (between 9% and 13% of total RBCs) and 3 weeks postinjection. We found differences between lipids in terms of both percent RBC and percent MFI (Figure 3C,D). Thus, DiI-C18, DiI-C18:2, DiI-PEG3400Mtz, and Cy3-cholesterol RBCs exhibited the longest circulation and retention. DiI-C18 RBCs had ≈40 days circulation span and only about 33% decrease in MFI at Day 20. DiI-C18-PEG-MTz and DiI-C18:2 RBCs showed somewhat shorter circulation span of ≈30 days and similar decrease in MFI as DiI-C18 at Day 20. Cy3-cholesterol RBCs had ≈30 days circulation span, but MFI dropped below 35% at Day 22. Shorter DiI-C12 RBCs had a circulation span of ≈20 days, and much faster decrease in MFI than DiI-C18 and Cy3-cholesterol (Figure 3C,D). The circulation of Cy3-C12 RBCs and Cy3-DSPE RBCs was the shortest, and both disappeared within a few days postinjection (Figure 3C,D). Because of rapid clearance, it was not possible to reliably measure MFI for Cy3-DSPE, but MFI for Cy3-C12 dropped by 85% at Day 3 (Figure 3D).

We next questioned whether the lipid retention and RBC longevity are prolonged in the immunodeficient host. NOD-SCID-gamma (NSG) mice are severely immunodeficient with dysfunctional macrophage, adoptive, and innate (e.g., complement) responses.^[14]^ We injected NSG mice with DiI-C12 RBCs that showed fast removal of the lipid and short half life in BALB/c mice. DiI-C12 RBCs demonstrated similar life span and similar change in MFI to BALB/c mice (Figure 3E,F), suggesting that most of the elimination of lipids from RBCs is not mediated by the immune system.

**Figure 4.**
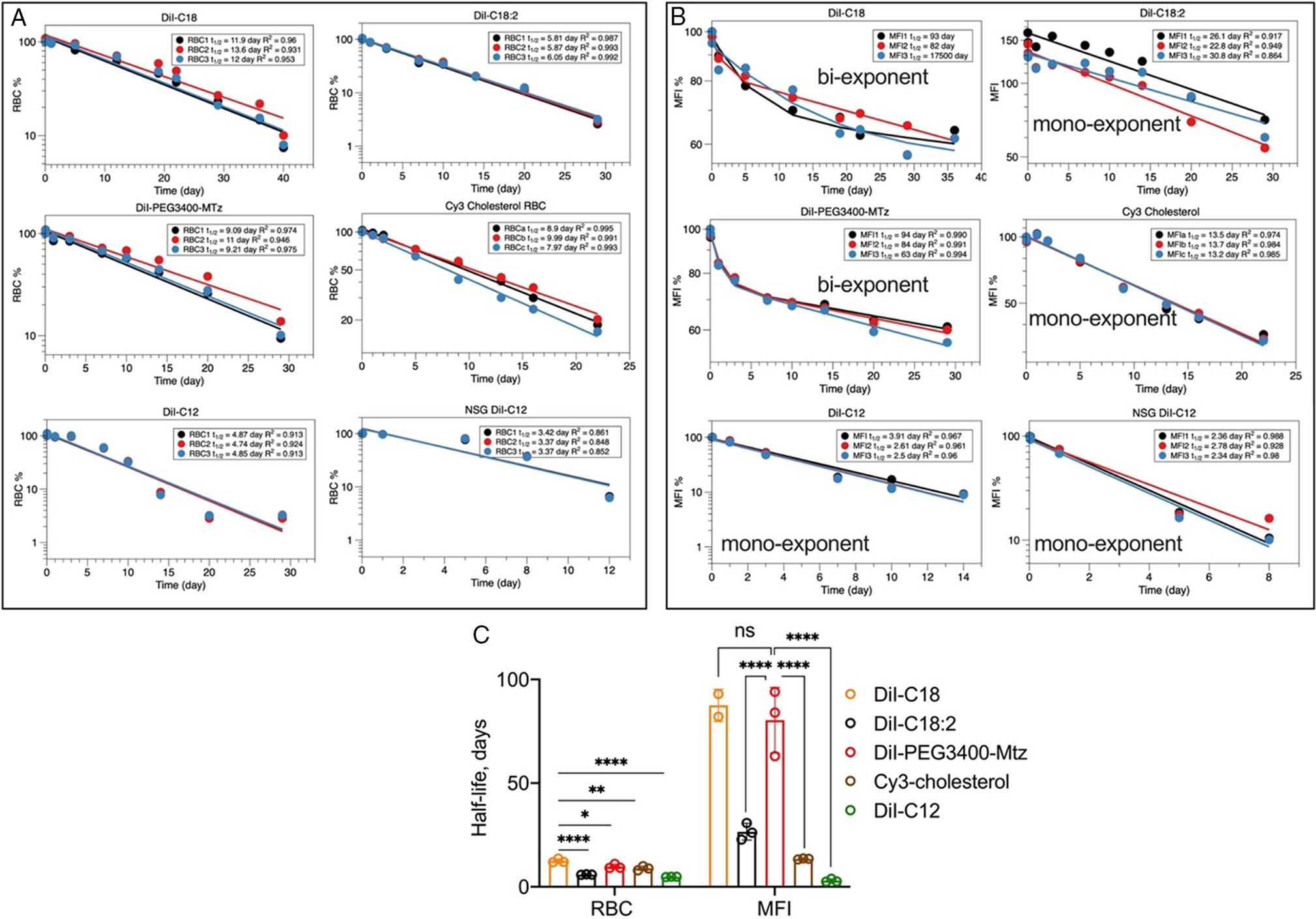
Pharmacokinetic analysis of data in Figure 3. A) One-compartment analysis of percent-labeled RBCs. B) % MFI based on extrapolation because some of the derivatives did not reach 50% of the initial level. DiI-C18 and DiI-PEG3400Mtz MFI values were best fitted with the two-compartment model; other were fitted with the one-compartment model. For DiI-C18, one of the mice did not produce a good fit. C) Summary of the analysis. Note that for some of the derivatives, the MFI half-life is much longer than the RBC half-life. DiI-C18 and DiI-PEG3400Mtz RBCs showed the longest half-life and the best stability in the membrane. *P*-value: ****<0.0001; ***<0.001,**<0.01,*0.05; one-way ANOVA with multiple comparisons.

Pharmacokinetic analysis of percent-labeled RBCs (Figure 4A, C) showed monoexponential decay, with DiI-C18 having the longest half life (12.5 days), followed by DiI-PEG3400Mtz (9.7 days), Cy3-cholesterol (9.2 days), DiI-C18:2 (5.9 days), and DiI-C12 (4.9 days in BALB/c, 3.6 days in NSG). The analysis of MFI half life (Figure 4B,C) showed either mono- or biexponential decay, with the estimated (extrapolated) terminal half life of over 80 days for DiI-C18 and DiI-PEG3400Mtz, and 25.6 days for DiI-C18:2, which is much longer than the RBC half life. DiI-C12 and Cy3-cholesterol MFI half lives were significantly shorter (DiI-C12: 3 days in BALB/C, 2.5 days in NSG; Cy3-cholesterol: 12.5 days).

**Figure 5.**
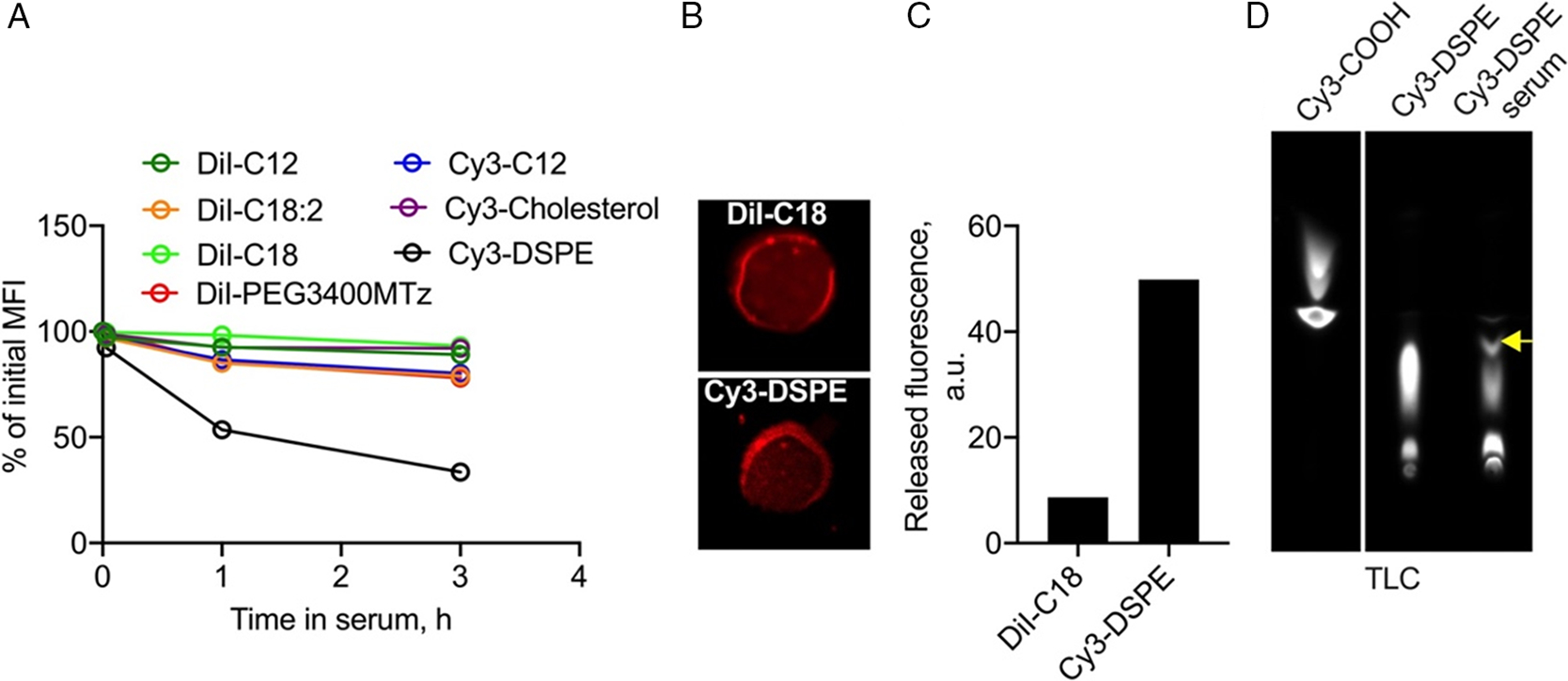
Stability of lipids in RBCs in vitro. A) Mouse RBCs labeled with lipid derivatives were incubated in plain BALB/c serum at 37 °C, and the release of the dye (percent of initial MFI) was monitored. Most of the lipids were stable at 3 h except Cy3-DSPE. B) Confocal microscopy shows mostly homogenous distribution of DiI-C18 and Cy3-DSPE in the membrane. C) Fluorescence released from labeled RBCs in serum at 3 h postincubation. D) TLC analysis of serum supernatant after incubation of Cy3-DSPE RBC for 3 h. Pure Cy3-COOH and Cy3-DSPE were run in parallel. Some degradation could be detected (arrow).

The nature of lipid linker affects in vivo retention. Thus, RBCs labeled with diacyl glycerol derivative Cy3-C12 and phospholipid Cy3-DSPE showed much faster removal than stable DiI-C12. To compare the stability of different lipids in vitro, we measured the fluorescence of labeled RBCs after incubation in mouse serum for 3 h. According to Figure 5A, DiI-C18, DiI-C18:2, DiI-PEG3400Mtz, DiI-C12, Cy3-C12, and Cy3-cholesterol RBCs showed less than 15% loss in MFI at 3 h. At the same time, Cy3-DSPE RBCs showed over 60% loss of MFI. Confocal microscopy showed that Cy3-DSPE and DiI-C18 had similar uniform labeling of RBCs prior to incubation in serum (Figure 5B). After 3 h incubation in serum, there was five times more fluorescence released in serum from Cy3-DSPE RBCs than from DiI-C18 RBCs (Figure 5C). Thin layer chromatography (TLC) analysis showed the presence of intact Cy3-DSPE along with some degradation products (Figure 5D). While 3 h time incubation is shorter than the in vivo longevity of some of the lipids (Figure 3B), the data indicate the removal of the phospholipid from RBC membrane through interaction with serum.

### 2.2. Immune Recognition of the Modified RBCs

**Figure 6.**
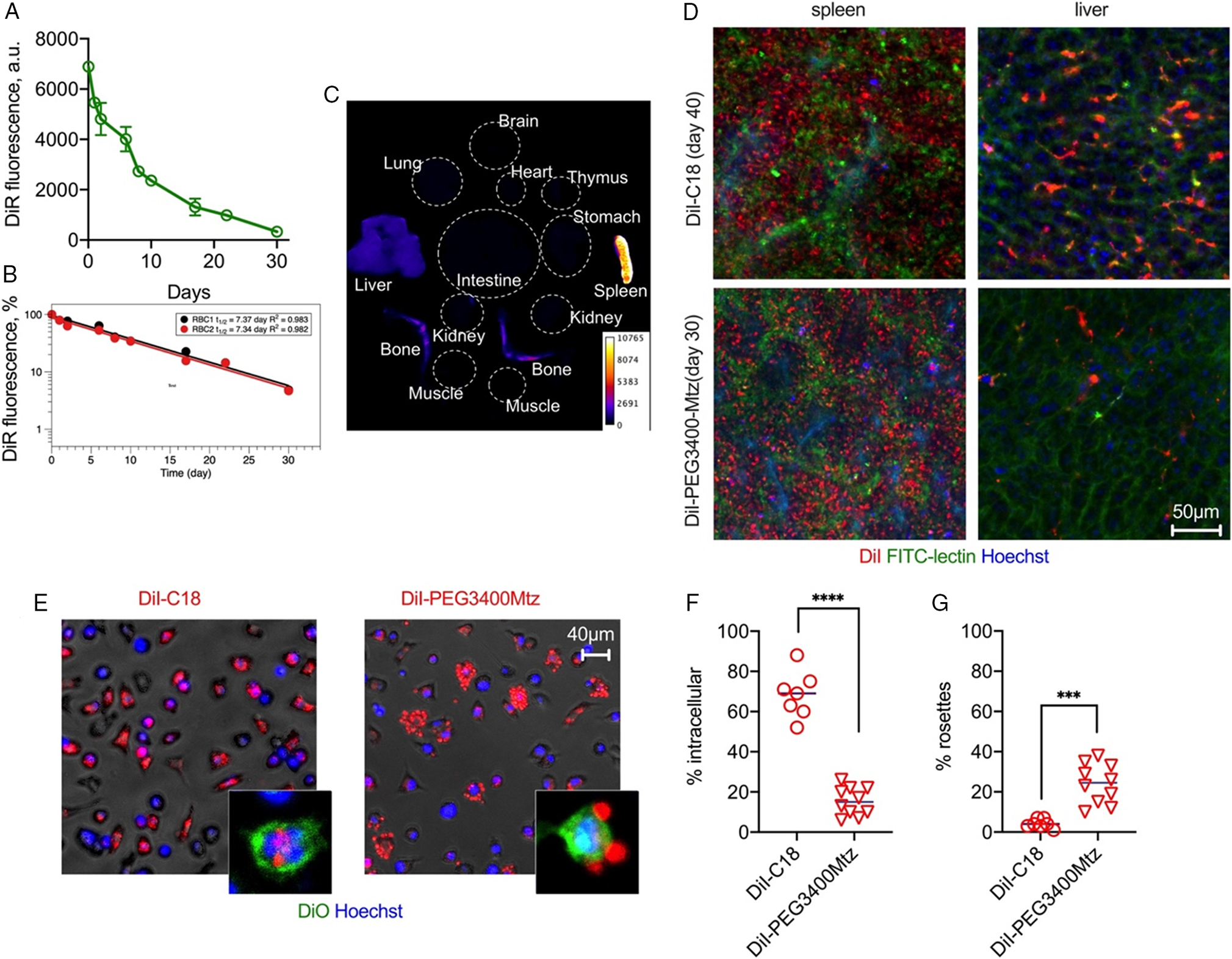
Biodistribution and immune recognition of RBCs. A) RBCs labeled with DiR were injected in BALB/c mice and blood fluorescence was monitored with NIR scanner Li-COR Odyssey. B) One-compartment pharmacokinetic analysis shows long half-life (*n* = 2 mice). C) Organ biodistribution of DiR fluorescence (pseudocolored) shows predominantly spleen and some liver and bone marrow accumulation. D) Confocal microscopy images of fresh livers and spleens of mice injected with DiI-C18 and DiI-PEG3400Mtz (after in vivo labeling of blood vessels and nuclei with FITC-lectin and Hoechst). The lipid accumulates in extrasinusoidal cells in the spleen and predominantly in sinusoidal cells in the liver (i.e., endothelium and Kupffer cells). DiI-PEG3400Mtz showed low accumulation in the liver. E) RBCs were incubated with fresh peritoneal macrophages for 24 h. DiI-C18 RBCs show mostly intracellular uptake, whereas DiI-PEG3400Mtz RBCs show mostly extracellular rosettes. Insets show confocal images of DiO-labeled macrophages to demonstrate intracellular versus extracellular localization. F) Quantification of percent cells (per field) that contain intracellular DiI. G) Quantification of percent cells (per field) that contain DiI+ rosettes. *P*-value: ****0.0001; ***<0.001 2-sided *t*-test, alpha 0.05.

We next asked which organs mediate the clearance of modified RBCs. Because ex vivo imaging at the Cy3 wavelength is challenging, we injected RBCs labeled with near-infrared indocarbocyanine lipid DiR-C18. We determined the levels of DiR-C18 RBCs in vivo by dotting blood and measuring total near-infrared (NIR) fluorescence with highly sensitive Li-COR Odyssey scanner (Figure 6A). DiR-C18 RBCs showed in vivo life span of over 30 days, and terminal half life of 7.3 days (Figure 6B), similar to that of DiI-C18. NIR imaging of organs 30 days postinjection of DiR-C18 RBCs showed predominant accumulation in the spleen, with minor accumulation in the liver and bone marrow (Figure 6C). To determine the location of the label in the clearance organs, we imaged freshly excised livers and spleens of BALB/c mice injected with DiI-C18 RBCs and DiI-PEG3400Mtz RBCs (Day 40 and Day 29, respectively) with confocal microscope. In all groups, there was a predominant accumulation of Cy3 signal in extrasinusoidal spleen cells and some accumulation in the liver sinusoids, (Figure 6D). Notably, there was evidence of fluorescence transfer to the sinusoidal endothelium in the liver (Figure 6D, upper right). While DiI-PEG3400Mtz did not reduce the accumulation in the spleen as compared with parent DiI-C18, we observed decreased accumulation in the liver (Figure 6D, lower right). To test if PEGylated RBCs are less prone to macrophage recognition, we incubated DiI-PEG3400Mtz RBCs and DiI-C18 RBCs with fresh mouse peritoneal macrophages for 24 h and studied the uptake by fluorescent microscopy. We found significantly fewer cells with intracellular Cy3 fluorescence in the DiI-PEG3400Mtz group (Figure 6E,F), but significantly more RBC rosettes around macrophages (Figure 6E,G), suggesting reduced internalization of PEGylated RBCs. Collectively, these data suggest that while PEGylation does not prevent the clearance by the spleen, it prevents the uptake by macrophages in the liver and in vitro.

**Figure 7.**
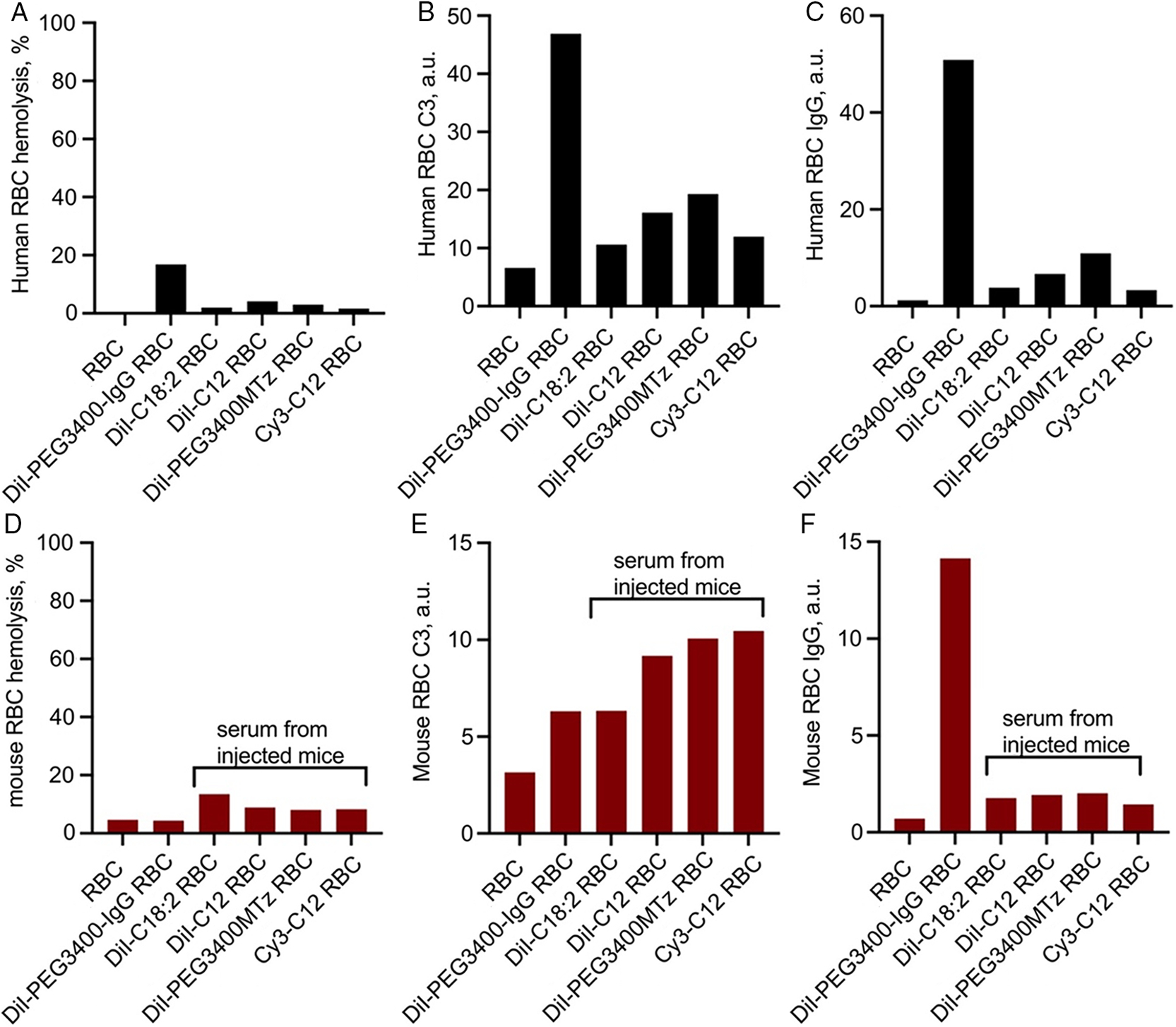
Hemolysis, complement opsonization, and IgG binding. Unlabeled or labeled RBCs were incubated in autologous human lepirudin plasma, or in mouse sera obtained from naïve BALB/c mice, or from mice injected with the corresponding RBCs as noted (sera were obtained on the last day of the experiment in Figure 3A). RBCs modified with DiI-PEG3400Mtz/IgG-TCO were used as a positive control in all studies. A–C) Human RBC hemolysis, C3 deposition, and IgG deposition, respectively, in human plasma. D–F) Mouse RBC hemolysis, C3 deposition, and IgG deposition, respectively, in mouse sera. Hemolysis was expressed as a percentage of fully hemolyzed RBCs. All other values are absolute fluorescence units of the dot-blot immunoassay (Experimental Section) and cannot be compared between human and mouse C3/IgG. There was a strong enhancement in hemolysis and C3 binding for IgG-modified RBCs in human plasma, but not in mouse sera. Some derivatives showed enhanced C3 and antibody response in both human plasma and mouse sera, but only human plasma showed correlation between bound IgG and complement opsonization/hemolysis.

Finally, we measured hemolysis, IgG binding, and complement C3 deposition on DiI-C12, Cy3-C12, DiI-C18:2, and DiI-PEG3400Mtz RBCs in autologous human lepirudin plasma and in mouse sera collected from mice injected with respective labeled RBCs (experiment in Figure 3; sera collected postmortem at D20, D20, D29, D29, respectively). For a positive control, we reacted DiI-PEG3400Mtz RBCs with human or mouse IgG-TCO. This two-step reaction results in over 100 000 IgG molecules per RBC,^[15]^ and these RBCs have a much shorter half life than DiI-PEG3400MTz RBCs.^[10]^ According to Figure 7A–C, “positive control” IgG-modified RBCs exhibited significant hemolysis, C3 opsonization, and IgG binding, whereas other modified RBCs showed much lower hemolysis, C3 opsonization, and IgG binding. Interestingly, DiI-PEG3400Mtz RBCs showed higher level of C3 and IgG deposition than other derivatives. For mouse RBCs, the relationship between hemolysis, C3, and IgG deposition was less clear. Thus, all derivatives showed elevated binding of IgG, but there was no correlation with hemolysis. Notably, DiI-PEG3400Mtz RBCs showed only a modest IgG binding, suggesting low antibody response to PEGylated RBCs in mice, as suggested before.^[13b]^

## 3. Discussion

The premise of this work was to understand the role of lipid structure in both the incorporation efficiency and the retention in the RBC membrane. We used a library of lipids with the same Cy3 headgroup to facilitate measurements by flow cytometry. Albeit fluorescence is not as quantitative tool as radioactive labeling^[5]^ and could be subject to quenching; it is commonly accepted for measurements of RBC longevity by flow cytometry^[12]^ and enables analysis of the percentage of labeled RBCs and their mean fluorescence. While our experiments did not reveal a general rule regulating in vitro labeling efficiency, short-chain derivatives promoted more efficient labeling of human RBCs than long-chain derivatives. On the other hand, some long-chain lipids showed better human RBC labeling efficiency than others. For example, Cy3-DSPE showed more efficient labeling than DiI-C18 (100% vs 28%), and DiI-PEG3400Mtz showed more efficient labeling than DiI-PEG5000 and DiI-amine. These results suggest that interactions between the lipid headgroup and the RBC membrane components could also determine the labeling efficiency. Propensity of lipid to form supramolecular assemblies could play a role in the ability to fuse with the membrane. Indeed, our previous study suggested that DiI micelle disassembly could be important for RBC incorporation.^[10]^ Therefore, lipids that have higher critical micelle concentration could more easily interact with the RBC membrane. Mouse RBCs showed better labeling efficiency than human RBCs, both in terms of MFI and percent labeling, which could be due to differences in membrane composition, physical properties, surface area, and metabolism between mouse and human RBCs^[16]^ or differences in the labeling medium (anticoagulant citrate dextrose [ACD] buffer for human RBCs, 1% BSA/PBS for mouse RBCs).

In vivo retention of the anchor was often in inverse relationship to in vitro labeling efficiency. Thus, DiI derivatives with long dialkyl chains or cholesterol exhibited ultralong retention in vivo, whereas Cy3 derivatives with ester bond, especially Cy3-DSPE, were unstable and quickly removed from RBCs. Our short-term serum incubation experiment suggests that lipid removal by serum could play some role in the loss in vivo, for example, due to transfer to albumin and lipoproteins. It is also possible that incomplete insertion into the membrane makes the ester bond more exposed to esterases. In case of DiI-PEG3400Mtz and DiI-C18, the estimated half life of the anchor was much longer than the reported 24-day half life of mouse RBCs.^[12,17]^ The half life of RBCs even in the case of DiI-C18 was shorter than 24 days, likely due to elimination of some damaged RBCs, as well as elimination of the label, leading to the inability to detect RBCs with flow cytometer. The loss of the label as one of the reasons for “apparent elimination” is indirectly supported by DiI-C12 and Cy3-cholesterol, for which the RBC half life closely matched the anchor half life. Most likely, the combined effect of the anchor on immune recognition and malleability of modified RBCs, as well as the removal of the label, result in dramatic differences in the circulation half life. PEGylation seems to have an additional effect on immunocompatibility of RBCs by decreasing C3 deposition and macrophage recognition, which could have an application for preparation of bioinert erythrocytes, for example, for production of universal blood. At the same time, the effect of PEGylation on hemolysis, complement activation, and antibody response is not clear. Previous study suggested no binding of anti-PEG antibodies to PEGylated RBCs in mouse and human serum,^[13b]^ while we found a small increase, which was PEG independent and IgG independent in mouse serum. While the translational significance of these finding remains to be elucidated, it appears that in vivo longevity of modified RBCs does not fully correlate with hemolysis, IgG binding, and C3 opsonization determined in vitro.

In summary, besides the practical aspect of RBC membrane derivatization for drug delivery and imaging, due to RBCs being long circulating “cells” and the ease of in vivo sampling, this study provides basic understanding on retention of lipids in the cell membrane under in vivo conditions.

## 4. Experimental Section

### Materials:

Fatty acids were from TCI America (Portland, OR, USA), PEG was from Laysan Bio (Arab, AL, USA), and MTz-NHS was from Click Chemistry Tools. DiI-C18:2, and DiI-C16, DiI-C12 were from ThermoFisher (Waltham, MA, USA). DiI-C18 (1,1′-Dioctadecyl-3,3,3′,3′-Tetramethylindotricarbocyanine Perchlorate) and DiR (1,1′-Dioctadecyl-3,3,3′,3′-Tetramethylindotricarbocyanine Iodide) were from Biotium (Hayward, CA, USA). DiI-NH_2_ was synthesized by a previously reported method.^[10]^ All the lipids were stored as 1 mM stocks in ethanol. Anticoagulant Citrate Dextrose (ACD) buffer was obtained from the Children’s Hospital Colorado Blood Donation Center and kept sterile before use. Goat IgG fraction to mouse C3 and human C3 (Catalog No. 55 463 and 55 033, respectively) was from MP Biomedicals (Solon, OH, USA). ChromPure human and mouse IgG was from Jackson Immuno Research (West Grove, PA, USA). Secondary antibodies IRDye 80°CW labeled donkey antigoat, goat antihuman, and goat antimouse IgG were from LI-COR Biosciences (Lincoln, NE, USA).

### Lipid Synthesis:

Scheme 1–5

### Synthesis of 2a-e:

DCC (3 eq.) in CH_2_Cl_2_ (5 mL) were added to a solution of *tert*-butyl (2,3-dihydroxypropyl)carbamate (1 eq). Fatty acid (3 eq.) and a catalytic amount of DMAP 0.1 eq.) dissolved in dry CH_2_Cl_2_ (10 mL) in nitrogen atmosphere. Stirring of the resulting mixture was continued for 24 h at room temperature, and the solvent was then evaporated under reduced pressure. The obtained product was purified by column chromatography (CH_2_Cl_2_/MeOH, 100/1). The product was obtained as a white powder. **2a**: Yield 68.3%. ^1^H NMR (400 MHz, CDCl_3_); δ 5.08 (m, 1H, CH), 4.24 (dd, 1H, CH_2_), 4.10 (dd, 1H, CH_2_), 3.35 (m, 2H, CH_2_), 2.30 (q, 4H, CH_2_), 1.60 (m, 4H, CH_2_), 1.42 (s, 9H, CH_3_), 1.25 (m, 32H, CH_2_), 0.87 (t, 6H, CH_3_).

**2b:** Yield 71.9%. ^1^H NMR (400 MHz, CDCl_3_); δ 5.08 (m, 1H, CH), 4.24 (dd, 1H, CH_2_), 4.12 (dd, 1H, CH_2_), 3.36 (m, 2H, CH_2_), 2.30 (q, 4H, CH_2_), 1.61 (m, 4H, CH_2_), 1.43 (s, 9H, CH_3_), 1.26 (m, 40H, CH_2_), 0.87 (t, 6H, CH_3_).

**2c:** Yield 68.3%. ^1^H NMR (400 MHz, CDCl_3_); δ 5.09 (m, 1H, CH), 4.29 (dd, 1H, CH_2_), 4.11 (dd, 1H, CH_2_), 3.34 (m, 2H, CH_2_), 2.34 (m, 4H, CH_2_), 1.61 (m, 4H, CH_2_), 1.43 (s, 9H, CH_3_), 1.25 (m, 48H, CH_2_), 0.88 (t, 6H, CH_3_).

**2d:** Yield 63.3%. ^1^H NMR (400 MHz, CDCl_3_); δ 5.34 (m, 4H, =CH, CH_2_), 5.08 (m, 1H, CH), 4.28 (dd, 1H, CH_2_), 4.11 (dd, 1H, CH_2_), 3.35 (m, 2H, CH_2_), 2.31 (m, 4H, CH_2_), 2.01 (m, 8H, CH_2_), 1.62 (m, 4H, CH_2_), 1.43 (s, 9H, CH_3_), 1.26 (m, 48H, CH_2_), 0.88 (t, 6H, CH_3_).

**2e:** Yield 85.1%. ^1^H NMR (400 MHz, CDCl_3_); δ 5.08 (m, 1H, CH), 4.25 (dd, 1H, CH_2_), 4.13 (dd, 1H, CH_2_), 3.37 (m, 2H, CH_2_), 2.31 (m, 4H, CH_2_), 1.62 (m, 4H, CH_2_), 1.43 (s, 9H, CH_3_), 1.25 (m, 52H, CH_2_), 0.88 (t, 6H, CH_3_).

### Synthesis of 3a-e:

Compound **2a-e** dissolved in CH_2_Cl_2_ with trifluoracetic acid (25%) in (10 mL) in nitrogen atmosphere. Stirring of the resulting mixture was continued for 30 min at room temperature, and the solvent was then evaporated under reduced pressure. The obtained product was purified by column chromatography (CH_2_Cl_2_/MeOH, 100/1). The product was obtained as a white powder.

**3a:** Yield 81.6 %. ^1^H NMR (400 MHz, DMSO-*d*_6_); δ 8.16, 5.57 (bs, 2H, NH_2_), 5.18 (m, 1H, CH), 4.29 (dd, 1H, CH_2_), 4.04 (dd, 1H, CH_2_), 3.13 (dd, 1H, CH_2_), 3.06 (dd, 1H, CH_2_), 2.26 (q, 4H, CH_2_), 1.49 (m, 4H, CH_2_), 1.23 (m, 32H, CH_2_), 0.84 (t, 6H, CH_3_).

**3b:** Yield 67.3 %. ^1^H NMR (400 MHz, CDCl_3_); δ 7.72 (bs, 2H, NH_2_), 5.28 (m, 1H, CH), 4.29 (m, 2H, CH_2_), 4.09 (m, 1H, CH), 3.97 (m, 2H, CH_2_), 2.42 (t, 2H, CH_2_), 2.29 (t, 2H, CH_2_), 1.63 (m, 4H, CH_2_), 1.23 (m, 40H, CH_2_), 0.86 (t, 6H, CH_3_).

**3c:** Yield 78.1%. ^1^H NMR (400 MHz, CDCl_3_); δ 5.62 (bs, 2H, NH_2_), 5.11 (m, 1H, CH), 4.29 (m, 1H, CH_2_), 4.13 (m, 1H, CH_2_), 3.37 (m, 2H, CH_2_), 2.34 (m, 4H, CH_2_), 1.63 (m, 4H, CH_2_), 1.25 (m, 48H, CH_2_), 0.88 (t, 6H, CH_3_).

**3d:** Yield 73.1%. ^1^H NMR (400 MHz, CDCl_3_); δ 7.97 (bs, 2H, NH_2_), 5.34 (m, 4H, =CH, CH_2_), 5.26 (m, 1H, CH), 4.30 (dd, 1H, CH_2_), 4.13 (dd, 1H, CH_2_), 3.23 (m, 2H, CH_2_), 2.32 (m, 4H, CH_2_), 2.01 (m, 8H, CH_2_), 1.60 (m, 4H, CH_2_), 1.26 (m, 40H, CH_2_), 0.88 (t, 6H, CH_3_).

**3e:** Yield 85.1%. ^1^H NMR (400 MHz, CDCl_3_); δ 5.08 (m, 1H, CH), 4.25 (dd, 1H, CH_2_), 4.13 (dd, 1H, CH_2_), 3.37 (m, 2H, CH_2_), 2.31 (m, 4H, CH_2_), 1.62 (m, 4H, CH_2_), 1.25 (m, 52H, CH_2_), 0.88 (t, 6H, CH_3_).

### Synthesis of 4a-e:

Compound 3a-e (1 eq), Cy3-COOH (1.1 eq.), HBTU (3 eq), and DIEA (3 eq.) dissolved in dry DMF (10 mL) in nitrogen atmosphere. Stirring of the resulting mixture was continued for 24 h at room temperature, and the solvent was then evaporated under reduced pressure. The obtained product was purified by column chromatography (CH_2_Cl_2_/MeOH, 100/1). The product was obtained as a white powder.

**C12-Cy3, 4a:** Yield 88.3%. ^1^H NMR (400 MHz, CDCl_3_); δ 8.43 (t, 1H, =CH), 7.39 (m, 4H, Ar-H), 7.31(m, 2H, Ar-H), 7.15 (m, 2H, Ar-H), 6.38 (m, 2H, =CH), 5.16 (m, 1H, CH), 4.28 (m, 1H, CH_2_), 4.11 (m, 1H, CH_2_), 4.02 (m, 2H, CH_2_), 3.65 (s, 3H, CH_3_), 3.54 (m, 1H, CH_2_), 3.45 (m, 1H, CH_2_), 2.34 (q, 4H, CH_2_), 1.82 (m, 2H, CH_2_), 1.74 (s, 12H, CH_3_), 1.59 (m, 4H, CH_2_), 1.51 (m, 2H, CH_2_), 1.26 (m, 32H, CH_2_), 0.88 (t, 6H, CH_3_).

**C14-Cy3, 4b:** Yield 68.3%. ^1^H NMR (400 MHz, CDCl_3_); δ 8.39 (t, 1H, =CH), 7.38 (m, 4H, Ar-H), 7.28 (m, 2H, Ar-H), 7.14 (m, 2H, Ar-H), 6.67 dd, 2H, =CH), 5.13 (m, 1H, CH), 4.28 (m, 1H, CH_2_), 4.02 (m, 1H, CH_2_), 4.02 (m, 2H, CH_2_), 3.65 (s, 3H, CH_3_), 3.52 (m, 1H, CH_2_), 3.45 (m, 1H, CH_2_), 2.31 (m, 4H, CH_2_), 1.81 (m, 2H, CH_2_), 1.72 (s, 12H, CH_3_), 1.58 (m, 6H, CH_2_), 1.25 (m, 40H, CH_2_), 0.86 (t, 6H, CH_3_).

**C16-Cy3, 4c:** Yield 77.2 %. ^1^H NMR (400 MHz, CDCl_3_); δ 8.38 (t, 1H, =CH), 7.37 (m, 4H, Ar-H), 7.28 (m, 2H, Ar-H), 7.14 (m, 2H, Ar-H), 6.46 (m, 2H, =CH), 5.14 (m, 1H, CH), 4.26 (m, 1H, CH_2_), 4.02 (m, 3H, CH_2_), 3.66 (s, 3H, CH_3_), 3.52 (m, 2H, CH_2_), 2.39 (m, 6H, CH_2_), 1.79 (m, 2H, CH_2_), 1.72 (s, 12H, CH_3_), 1.57 (m, 4H, CH_2_), 1.50 (m, 2H, CH_2_), 1.39 (m, 2H, CH_2_), 1.23 (m, 48H, CH_2_), 0.87 (t, 6H, CH_3_).

**C18:1-Cy3, 4d:** Yield 53.8 %. ^1^H NMR (400 MHz, CDCl_3_); δ 8.39 (t, 1H, =CH), 7.37 (m, 4H, Ar-H), 7.28 (m, 2H, Ar-H), 7.15 (m, 2H, Ar-H), 6.54 (dd, 2H, =CH), 5.33 (m, 4H, =CH, CH_2_), 5.13 (m, 1H, CH), 4.28 (dd, 1H, CH_2_), 4.06 (m, 1H, CH_2_), 3.67 (s, 3H, CH_3_), 3.52 (m, 1H, CH_2_), 3.45 (m, 1H, CH_2_), 2.44 (m, 2H, CH_2_), 2.29 (m, 4H, CH_2_), 2.00 (m, 8H, CH_2_), 1.81 (m, 2H, CH_2_), 1.72 (s, 12H, CH_3_), 1.58 (m, 6H, CH_2_), 1.28 (m, 40H, CH_2_), 0.87 (t, 6H, CH_3_).

**C18-Cy3, 4e:** Yield 81.7 %. ^1^H NMR (400 MHz, CDCl_3_); δ 8.39 (t, 1H, =CH), 7.37 (m, 4H, Ar-H), 7.26 (m, 2H, Ar-H), 7.14 (m, 2H, Ar-H), 6.58 (m, 2H, =CH), 5.14 (m, 1H, CH), 4.25 (dd, 1H, CH_2_), 4.09 (m, 1H, CH_2_), 4.03 (m, 2H, CH_2_), 3.66 (s, 3H, CH_3_), 3.52 (m, 1H, CH_2_), 3.46 (m, 1H, CH_2_), 2.29 (m, 6H, CH_2_), 1.82 (m, 2H, CH_2_), 1.73 (s, 12H, CH_3_), 1.58 (m, 6H, CH_2_), 1.25 (m, 52H, CH_2_), 0.87 (t, 6H, CH_3_).

### Synthesis of VA-PEG3400-NHMTz:

A mixture of amine–PEG–valeric Acid (NH_2_-PEG3400-VA) (50 mg, 0.015 mmol, 1 eq.), methyl tetrazine NHS (7.21 mg 0.022 mmol, 1.5 eq.), and DIEA (8 μL, 0.044 mmol, 3 eq.) was stirred in DMF at room temperature for 4 h. The solvent was then evaporated under reduced pressure and the resulting dark pink residue was purified using prep HPLC and eluted with 40–50% methanol/water, to obtain VA-PEG3400-NHMTz as a pink solid. Yield 68.3%. ^1^H NMR (400 MHz, CDCl_3_); δ 9.98 (bs, 1H, NH), 8.51 (d, *J* = 8.3 Hz, 2H, Ar-H), 7.48 (d, *J* = 8.3 Hz, 2H, Ar-H), 3.38–3.81 (m, 330H, CH_2_) 3.05 (s, 3H, CH_3_), 2.31 (t, *J* = 7.2 Hz, 2H, CH_2_), 1.53–1.72 (m, 4H, CH_2_).

### Synthesis of DiI-PEG3400MTz:

A mixture of DiI-NH_2_ (5 mg, 0.0058 mmol, 1 eq.), VA-PEG3400-NHMTz (20.8 mg, 0.0058 mmol, 1 eq.), HBTU (3.3 mg, 0.0087 mmol, 1.5 eq.), and DIEA (3.2 μL, 0.017 mmol, 3 eq.) was stirred in DMF at room temperature for 12 h. The solvent was then evaporated under reduced pressure and the resulting dark red-orange residue was purified using prep HPLC and eluted with 70–80% methanol/water, to obtain DiI-PEG3400MTz as a dark red-orange solid. Yield. 83.5%. ^1^H NMR (400 MHz, CDCl_3_); δ 8.58 (d, *J* = 8.3 Hz, 2H, Ar-H), 7.75 (m, 2H, Ar-H), 7.65 (m, 2H, Ar-H), 7.56 (d, *J* = 8.3 Hz, 2H, Ar-H), 7.33–7.46 (m, 2H, Ar-H), 7.16–7.20 (m, 1H, Ar-H), 7.00–7.04 (m, 1H, Ar-H), 6.78–6.83 (m, 1H, CH), 6.55–6.63 (m, 1H, CH), 4.37–4.71 (m, 6H, CH_2_), 4.16–4.26 (m, 2H, CH_2_), 3.79–3.87 (m, 2H, CH_2_), 3.42–3.74 (m, 190H, CH_2_), 3.12 (s, 3H, CH_3_), 2.32–2.42 (m, 2H, CH_2_), 1.56–1.88 (m, 12H, CH_2_), 1.12–1.46 (m, 60H, CH_2_), 0.90 (t, *J* = 6.9 Hz, 6H, CH_3_).

### Synthesis of DiI-mPEG5000:

A mixture of DiI-NH_2_ (5 mg, 0.0058 mmol, 1 eq.), mPEG5000 (15, 20.8 mg, 0.0058 mmol, 1 eq.), HBTU (3.3 mg, 0.0087 mmol, 1.5 eq.) and DIEA (3.2 μL, 0.017 mmol, 3 eq.) was stirred in DMF at room temperature for 12 h. The solvent was then evaporated under reduced pressure and the resulting dark red-orange residue was purified using prep HPLC and eluted with 70–80% methanol/water, to obtain DiI-mPEG5000 as a dark red-orange solid. Yield. 83.5%. ^1^ H NMR (400 MHz, CDCl_3_); δ 7.78 (m, 2H, Ar-H), 7.6 (m, 2H, Ar-H), 7.28 (m, 2H, Ar-H), 7.17 (m, 1H, Ar-H), 7.01 (m, 1H, Ar-H), 6.76 (m, 1H, CH), 6.58 (m, 1H, CH), 4.37–4.71 (m, 6H, CH_2_), 3.8 (m, 2H, CH_2_), 3.61 (bs, OCH_2_), 2.38 (m, 4H, CH_2_), 1.74 (m, 12H, CH_2_), 1.26 (m, 60H, CH_2_), 0.90 (t, *J* = 6.9 Hz, 6H, CH_3_).

### Synthesis of Cy3-DSPE:

A mixture of DSPE–NH_2_ (2 mg, 0.0026 mmol, 1 eq.) Cy3 NHS (2.57 mg 0.004 mmol, 1.5 eq.), and DIEA (1.2 μL, 0.008 mmol, 3 eq.) was stirred in chlorform:methanol (9:1) at 50 °C for 2 h. The solvent was then evaporated under reduced pressure and the resulting dark pink residue was purified by preparatory HPLC (C18 column) and eluted with 70–80% methanol/water (0.1% TFA), to obtain Cy3-DSPE as a pink solid. The product was characterized by MALDI TOF mass spectrometry and was found to be 1186.70 Da (exact mass of BF_4_^−^ salt 1274.50 Da).

### Synthesis of Cy3-Cholesterol:

DCC (3 eq.) in CH_2_Cl_2_ (5 mL) was added to a solution of cholesterol (1 eq), Cy3-COOH (1.1 eq.), and a catalytic amount of DMAP (0.1 eq.) dissolved in dry CH_2_Cl_2_ (10 mL) in nitrogen atmosphere. Stirring of the resulting mixture was continued for 24 h at room temperature, and the solvent was then evaporated under reduced pressure. The obtained product was purified by column chromatography (CH_2_Cl_2_/MeOH, 100/1). The product was obtained as a pink solid (Yield 58%). ESI–MS, Calculated *m/z* = 825.63, Found *m/z* = 826.5 [M + H], 828.4 [M + 2] (exact mass of I^−^ salt 953.2 Da).

### RBC Labeling:

Fresh human RBCs were obtained from discarded leukodepletion filters after processing sodium citrate anticoagulated donor blood at the Children’s Hospital Colorado Blood Donation Center. Institutional review board approval was not required for discarded material and anonymous samples. RBCs were eluted from leukodepletion filters by applying ACD buffer in the direction of the flow and were used within 2 h after blood collection. Sodium EDTA-anticoagulated mouse blood was collected from female or male BALB/c and NSG mice (8–10 weeks of age) via cardiac puncture, according to the animal protocol approved by the University of Colorado IACUC). Human RBCs were washed in ACD buffer and mouse RBC were washed in 1% BSA/PBS at room temperature at 3,000 g, total 3 times. Erythrocyte suspension (≈10^10^/mL) was incubated with 25 μM lipids in ACD buffer (human RBCs) or 1% BSA/PBS (mouse RBCs) at 37 °C for 1 h and washed three times in ACD buffer or 1% BSA/PBS as described above. Labeling efficiency were determined by Guava easyCyte HT flow cytometer (Luminex Corp, Seattle, WA) as described later.

### In vivo Circulation and Biodistribution:

The University of Colorado Institutional Animal Care and Use Committee (IACUC) approved animal experiments (protocol 103 913(11)1D). Mice were treated according to regulations provided by the Office of Laboratory Animal Resources at the University of Colorado. BALB/c and NOD/LtSz-SCID IL2Rγc null (NSG) mice were bred in-house. Mice of 8–10 weeks age (males and females) were used for experiments. Mice were injected intravenously with ≈10^9^ RBCs (each strain was injected with RBCs derived from the same strain). At different time intervals, ≈20 μL of blood were obtained with heparinated haematocrit capillary (ThermoFisher) via periorbital plexus. RBCs were resuspended at ≈0.5 million mL^−1^ in 1%BSA/PBS and analyzed by Guava easyCyte HT flow cytometer (20 000 RBCs were counted after gating out platelets and leukocytes). The percentage of positive RBCs and mean fluorescence intensity (MFI) of labeled RBCs (if more than 1% of total RBCs) was determined using FlowJo software v.10 (BD Life Sciences, Ashland, OR). For pharmacokinetic analysis, the flow data were normalized to 100% injected dose (MFI and percent labeled RBCs at 1 min) and fit into mono or biexponential decay using the pharmacokinetic modeling software Boomer.^[21]^

When labeled RBCs reached <1% of total RBCs (usually 10% or less of the injected dose), mice were injected with FITC-lectin and Hoechst and sacrificed with carbon dioxide (CO_2_) followed by cardiac perfusion. Fresh livers and spleens were placed on glass slides and imaged with Nikon Eclipse AR1HD inverted confocal microscope with Plan Apo 10 objective as described.^[18]^ For imaging of biodistribution of DiR-labeled RBCs, main organs were arranged in a Petri dish.

### Lipid Stability in Mouse Serum:

Mouse RBC labeled with lipids were incubated in mouse serum at 37 °C for up to 3 h. Intact RBCs were removed by centrifugation at 3000 g for 10 min at room temperature. RBCs were washed three times in 1% BSA/PBS to remove the remaining serum, and the labeling (MFI) was determined by flow cytometry as described above. The supernatant was analyzed for fluoresce released from RBC by dotting 2 μL on 0.45 μm nitrocellulose membrane and scanning with a Bio-Rad gel imager for Cy3 fluorescence at 540 nm excitation/560 nm emission. For TLC, 10 parts of methanol were added to 1 part of serum, and the tubes were centrifuged at 500 g for 10 min to pellet the protein fraction. The methanol phase was carefully collected, applied on TLC Silica Gel 60 F254 (EMD Millipore), and separated on the mobile phase chloroform: methanol (9:1) with 0.1% trifluoroacetic acid. The plates were scanned with a Bio-Rad gel imager for Cy3 fluorescence.

### Uptake by Peritoneal Macrophages:

Fresh nonactivated macrophages were isolated and seeded in culture for 24 h in a 96-well culture-treated plate (Corning Inc.) as described.^[19]^ On the next day, labeled RBCs were added (≈1 × 10^5^ RBC/well) in triplicates, and macrophages were incubated for 24 h. In some experiments, PMF were prelabeled with 10 μM DiO for 2 h before addition of RBCs. After incubation, macrophages were washed with PBS 4 times to remove nonbound RBCs, fixed with 4% formalin, and stained with nuclear stain Hoechst. Cells were imaged under 100× magnification with Zeiss Axio Observer 5 epifluorescent microscope, and ≈7 microscopic fields were acquired. To verify RBC localization outside the cells, a Nikon Eclipse AR1HD inverted confocal microscope with 405, 488, 561, and 640 nm excitation lasers and corresponding emission filters was used. The percentage of DiI+ cells and rosette+ cells per field was determined manually and plotted with Prism.

### Hemolysis, IgG, and C3 Deposition on Human and Mouse RBCs:

Lepirudin–anticoagulated blood was obtained from healthy donors as described before and processed immediately.^[20]^ Blood was centrifuged at 3000 g for 25 min at 4 °C, plasma was collected, and kept on ice. Lepirudin plasma allows complement activation and was used with autologous RBCs in subsequent experiments. Buffy coats were aspirated, and RBCs were washed in ACD buffer and labeled with lipid derivatives as above. For a negative control, plain nonlabeled RBCs were used. For a positive control, RBCs were first labeled with DiI-PEG3400Mtz and then reacted with mouse or human IgG-TCO as described before^[15]^ to produce IgG-coated RBCs. All RBCs were washed once in PBS and were incubated in autologous lepirudin plasma at 37 °C for 1 h (1:4 RBC:plasma volume ratio). RBCs were pelleted by centrifugation at 3,000 g for 10 min at room temperature. The supernatant was collected, diluted with PBS, and the absorbance of hemoglobin was measured at 540 nm. The relative hemolysis was expressed as percent change from nonlabeled RBCs incubated in naïve serum. For C3/IgG detection by dot-blot immunoassay, RBC pellet was washed three times in 1% BSA/PBS to remove the remaining serum, resuspended in PBS, and 2 μL of sample was applied in triplicates on a 0.45 μm-pore nitrocellulose membrane (Bio-Rad). The membranes were blocked with 5% w/v milk and probed with anti-C3 antibody for 1 h at room temperature, washed, and then incubated with IRDye80°CW-labeled secondary antibody. For IgG detection, anti-human IgG IRDye 80°CW-labeled antibody was directly used. The membrane was scanned using Li-COR Odyssey infrared imager, and the integrated intensities of dots were determined from eight-bit grayscale images using Fiji software. The quantification data were plotted using Prism software v. 9.0 (GraphPad, San Diego, CA). The relative IgG and C3 binding was expressed as percent difference from nonlabeled RBCs incubated in naïve serum.

Mouse hemolysis, C3, and IgG binding experiments were performed exactly as above, except that serum collected from naïve BALB/c mice was used for incubation of negative or positive control RBCs, and serum from BALB/c mice injected with the corresponding labeled RBCs (on the last day of the experiment in Figure 3) was used for incubation with labeled RBCs.

## Figures and Tables

**Scheme 1.**
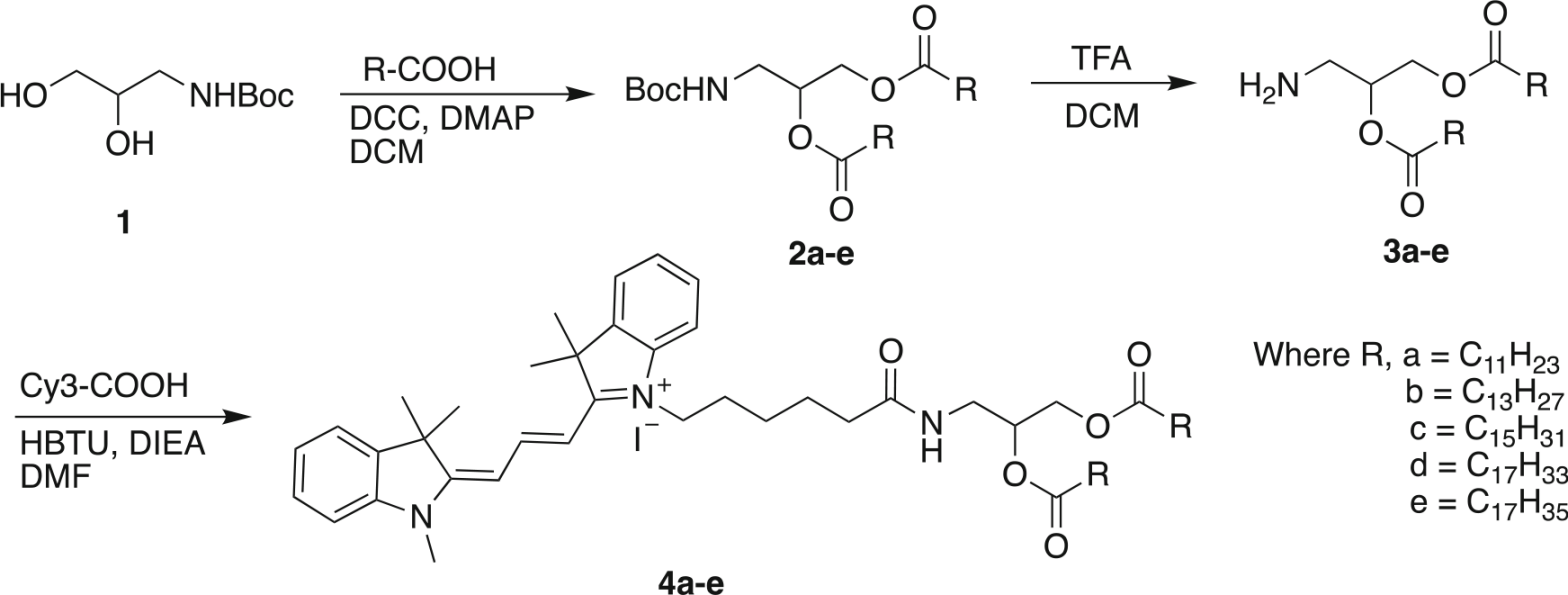
Synthesis of Cy3 analogues of fatty acids.

**Scheme 2.**
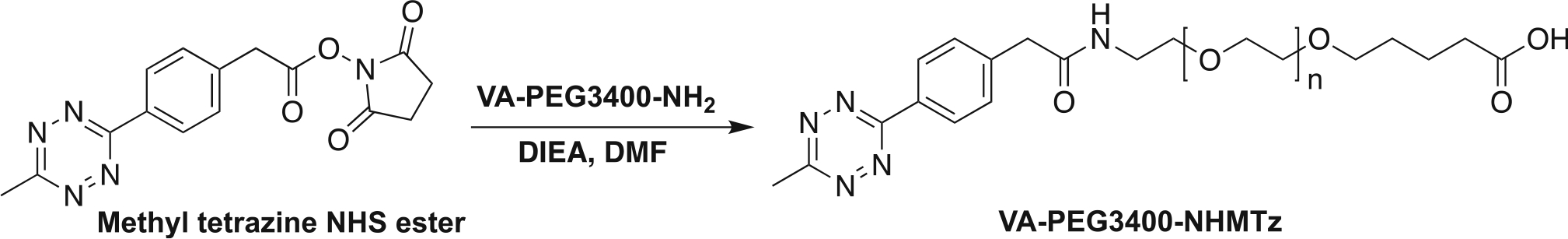
Synthesis of methyltetrazine (Mtz) PEG3400 Valeric acid.

**Scheme 3.**
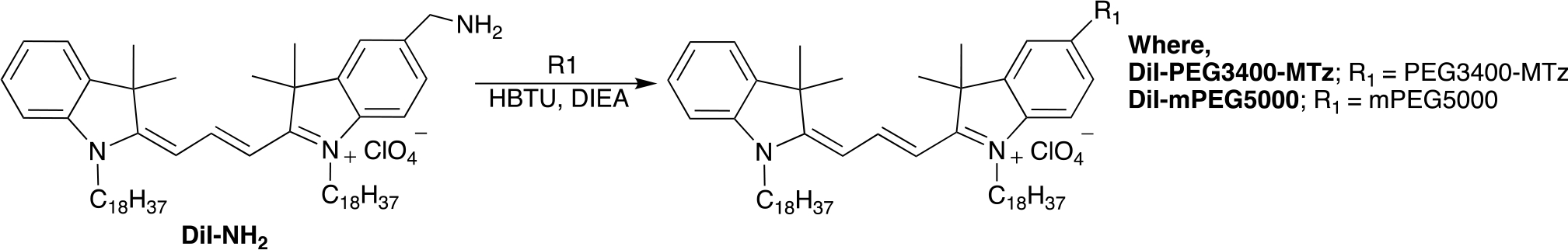
Synthesis of DiI analogues.

**Scheme 4.**
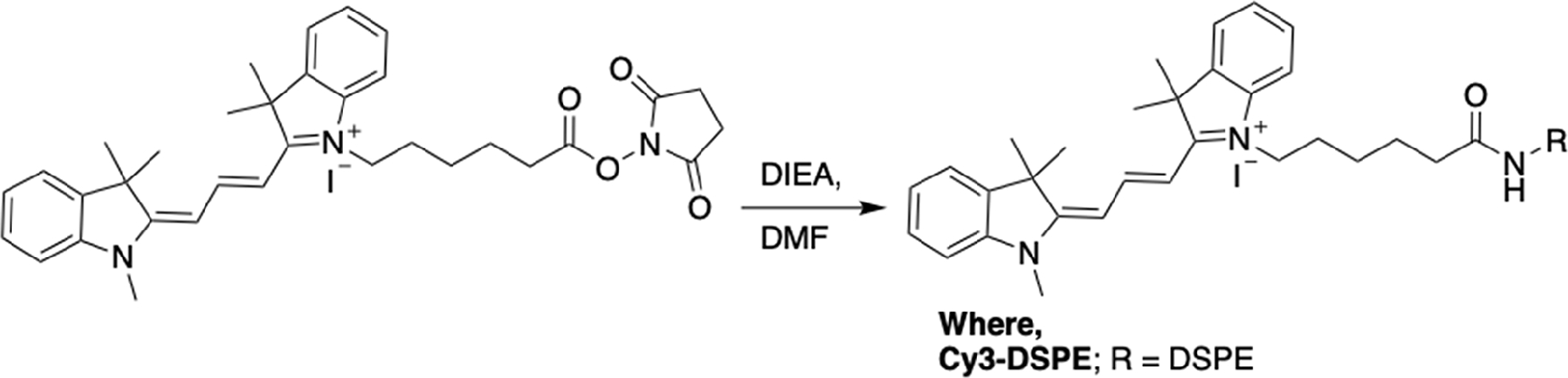
Synthesis of Cy3-DSPE analogues.

**Scheme 5.**
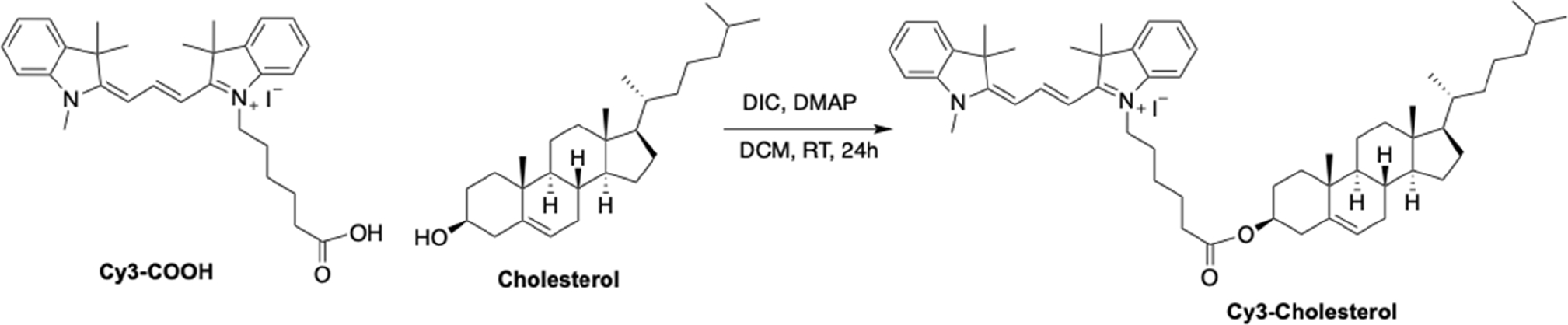
Synthesis of Cy3-Cholesterol analogues.

## Data Availability

The data that support the findings of this study are available from the corresponding author upon reasonable request.
